# Identification of systemic biomarkers and potential drug targets for age-related macular degeneration

**DOI:** 10.3389/fnagi.2024.1322519

**Published:** 2024-02-01

**Authors:** Shizhen Lei, Mang Hu, Zhongtao Wei

**Affiliations:** ^1^Department of Ophthalmology, Wuhan No. 1 Hospital, Tongji Medical College, Huazhong University of Science and Technology, Wuhan, Hubei, China; ^2^Wuhan Fourth Hospital, Wuhan, Hubei, China

**Keywords:** age-related macular degeneration, senescence, Mendelian, biomarker, drug target

## Abstract

**Purpose:**

Since age-related macular degeneration (AMD) is tightly associated with aging and cellular senescence, objective of this study was to investigate the association between plasma levels of senescence-related proteins (SRPs) and risk of AMD.

**Design:**

The whole study was based on two-sample Mendelian randomization (MR) analysis.

**Methods:**

For MR analysis, the primary approach for MR analysis was the inverse-variance weighted (IVW) method and the heterogeneity and pleiotropy of results were tested. The instrumental single-nucleotide polymorphisms (SNPs) associated with 110 SRPs were filtered and selected from a large genome-wide association study (GWAS) for plasma proteome involving 35,559 participants. The GWAS data of AMD was obtained from FinnGen consortium (6,157 AMD cases and 288,237 controls) and further validated by using data from UK Biobank consortium (3,553 AMD cases and 147,089 controls).

**Results:**

The MR results at both discovery and validation stages supported the causality (IVW-*P* < 0.00045) between plasma levels of 4 SRPs (C3b, CTNNB1, CCL1, and CCL3L1) and the risk of AMD and supported potential causality (IVW-*P* < 0.05) between other 10 SRPs and risk of AMD. No heterogeneity or pleiotropy in these results was detected.

**Conclusion:**

Our findings supported that high plasma levels of C3b, CTNNB1, CCL1, and CCL3L1 were associated with increased risk of AMD, thereby highlighting the role of systemic inflammation in AMD pathogenesis and providing the rationale for developing new preventative and therapeutic strategies.

## 1 Introduction

Age-related macular degeneration (AMD) is a neurodegenerative disease involving neuroretina and retinal pigment epithelium (RPE), thereby leading to visual impairment or even blindness (Wong et al., 2014). Studies have reported considerable health burden in patients with AMD, which mainly affects adults aged 40 years and older (Zhu et al., 2019). The number of patients with AMD is continuously increasing and estimated to be about 288 million worldwide by 2,040 (Congdon et al., 2004; Wong et al., 2014).

Intravitreal injection of anti-vascular endothelial growth factor (VEGF), such as ranibizumab (Blodi et al., 2023) and aflibercept (Wykoff et al., 2023), have been used to slow the progression of the neovascular or exudative subtype (nAMD or eAMD). However, for geographic or atrophic AMD, there is no effective treatment available. Therefore, it is necessary to identify risk factors for AMD to help prevent the incidence of AMD and alleviate the burden of this disease on public health. Some intraocular risk factors have been revealed, such as extracellular deposits (Chen et al., 2022). Notably, localized mechanistic studies in the eye have failed to fully elucidate pathogenesis of AMD and the systemic risk factors for AMD were still lacking, thereby limiting the interventions for reducing the risk of AMD.

As a hallmark of aging, cellular senescence is a significant contributor to aging and age-related diseases including Alzheimer’s disease (AD) (Holloway et al., 2023). Previous studies have suggested that oxidative stress, inflammation and RPE senescence may all play a critical role (Kauppinen et al., 2016) in AMD initiation and development. Notably, Saul et al. (2022) has identified a gene set (senescence-related genes, SRGs) for predicting senescence-associated pathways across tissues, which has been used and cited by many researches about age-related diseases (Doolittle et al., 2023; Farr, 2023).

Observational study and randomized clinical trial (RCT) are useful for identifying risk factors of diseases. However, observational studies are vulnerable to reverse causation, residual confounding, and selective bias (Davey Smith and Hemani, 2014). A RCT allows reliable and robust causal inferences to be drawn, but it is costly, time-consuming, and sometimes impractical to conduct one. Mendelian randomization (MR) approaches have opened up opportunities to assess and determine clinically associated characters for multiple diseases (Davey Smith and Hemani, 2014), which examines causal relationships between exposures and outcomes using genetic variants significantly associated with an exposure as instrumental variables. By using MR approaches, the defects of observational study and RCT can be nicely overcome (Davey Smith and Hemani, 2014). In addition, this approach has been used to explore risk factors of multiple eye disorders (Patasova et al., 2021; Choquet et al., 2022).

**FIGURE 1:**
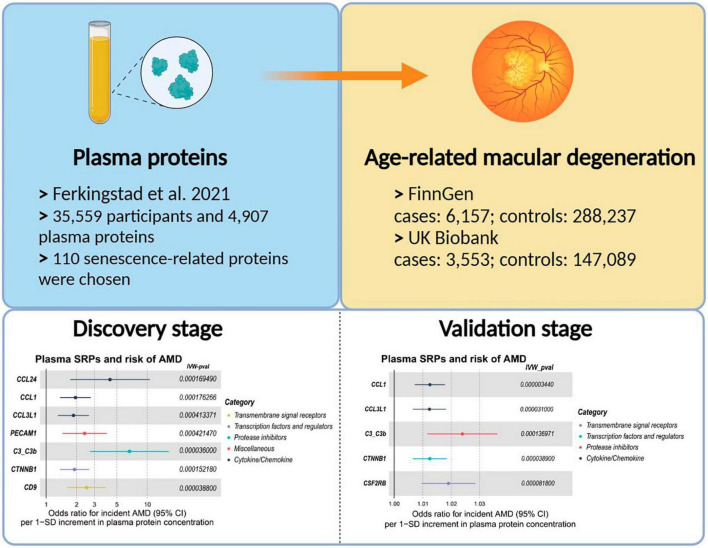
The summary of design and findings of this study.

In this study, we obtained the list of SRGs and explored the causality between the plasma levels of the proteins (senescence-related proteins, SRPs) encoded by these SRGs and the risk of AMD by two-sample MR analysis. The design and findings of this study were summarized in Figure 1. As a results, plasma levels of 4 SRPs (C3b, CCL1, CCL3L1, and CTNNB1) were identified and confirmed to be associated with risk of AMD. It is hoped that these 4 SRPs will serve as new drug targets for intervention.

## 2 Materials and methods

### 2.1 Theoretical foundation of MR analysis

**FIGURE 2:**
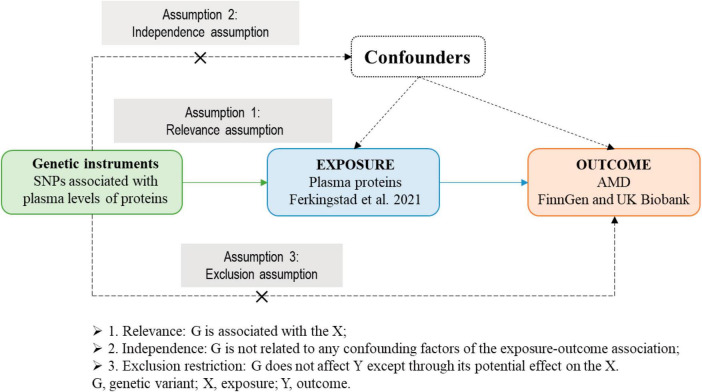
The theoretical basis and three basic assumptions of MR analysis. MR, Mendelian randomization.

The list of SRGs was obtained from Saul et al. (2022) (Supplementary Table 1). The theoretical basis and three basic assumptions of MR analysis (Emdin et al., 2017; Zheng et al., 2017) were shown in Figure 2. In this study, we implemented two-sample MR approaches to judge causation between plasma SRPs and AMD risk.

### 2.2 Data source for exposures and outcomes

**FIGURE 3:**
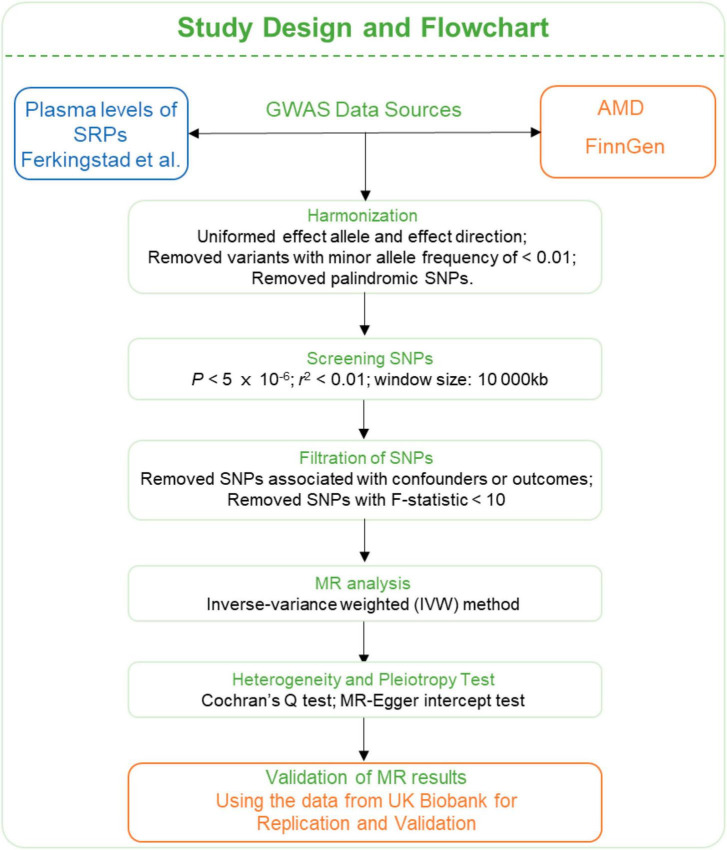
Flowchart of the MR analyses. MR: Mendelian randomization; SRPs, senescence-related proteins; AMD, age-related macular degeneration; GWAS, genome-wide association study; SNP, single nucleotide polymorphism; IVW, inverse-variance weighted.

The summary level genome-wide association studies (GWAS) data of SRPs were obtained from Ferkingstad et al. (2021), who have conducted a large-scale GWAS project on plasma proteome involving 35,559 participants and 4,907 plasma proteins. The GWAS data of AMD were obtained from FinnGen (6,157 cases and 288,237 controls) (Kurki et al., 2023) and UK Biobank consortium (3,553 cases and 147,089 controls) (Sudlow et al., 2015). The AMD cases were defined by H7 in International Classification of Disease-10 (ICD-10) and 3,625 in ICD-9. The FinnGen consortium data was used for discovery and data from UK Biobank consortium was for replication and validation. The flowchart of this study is presented in Figure 3.

### 2.3 Selection of genetic instruments

Instrumental variables (single-nucleotide polymorphisms, SNPs) were selected via the following criteria: (i) with genome-wide significance (*P* < 5 × 10^–6^) and (ii) pruned by linkage disequilibrium (*r*^2^ < 0.01 and within 10 000 kb from the index variant). PhenoScanner (Staley et al., 2016) is an online platform with comprehensive information about genotype-phenotype association. We examined whether the obtained instrumental SNPs were associated with the outcomes and the potential confounders and subsequently remove the associated ones.

### 2.4 Causality estimated by MR analysis

Mendelian randomization’s validity depends on the crucial assumption of no pleiotropy (Lawlor et al., 2008). Therefore, we used the random-effect inverse-variance weighted (IVW) method (Burgess et al., 2016) as the primary method and performed Cochran’s Q test and MR-Egger intercept test to evaluate the heterogeneity and detect pleiotropy (Bowden et al., 2019).

### 2.5 Identification of druggable targets

A list of druggable genes were obtained from Freshour et al. (2021) (Drug-Gene Interaction Database, DGIdb V.4.2.0^1^) (Supplementary Table 2). DGIdb provides information on drug-gene interactions and druggable genes from publications, databases and other web-based sources. We downloaded the “Categories Data” (released in February 2022), including all genes in the druggable categories in the DGIdb, from all sources.

### 2.6 Statistical analysis

We performed all the analyses in R (version 4.0.1) using the TwoSampleMR (Hemani et al., 2018) R packages. The code for MR analysis is accessible at https://mrcieu.github.io/TwoSampleMR/articles/index.html. All statistical tests are 2 sided. Results with IVW-*P* < 0.05 was considered nominally significant and IVW-*P* < 0.00045 was taken as statistically significant.

## 3 Results

### 3.1 MR results in the discovery stage

**FIGURE 4:**
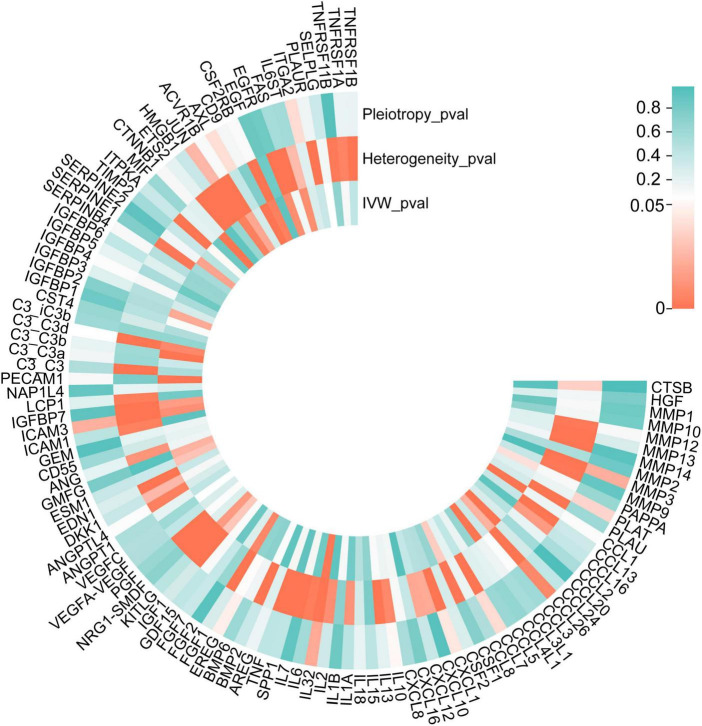
*P*-values of IVW, heterogeneity test, and pleiotropy test of SRPs and AMD risk in discovery stage. MR: Mendelian randomization; SRPs, senescence-related proteins; AMD, age-related macular degeneration; IVW, inverse-variance weighted.

**FIGURE 5:**
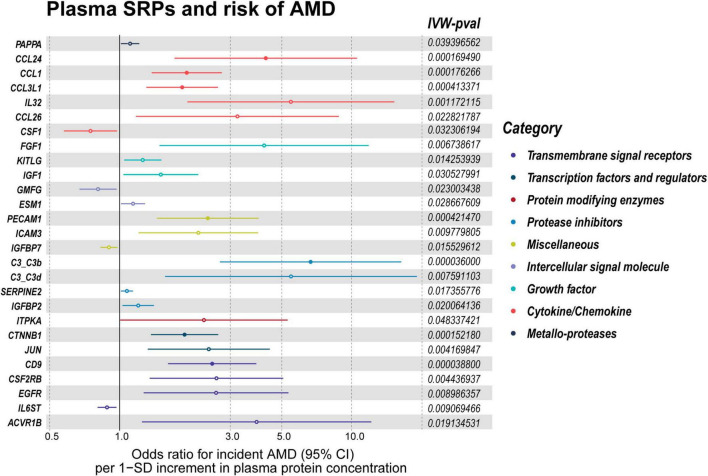
Forest plot of the MR results in discovery stage. MR: Mendelian randomization; SRPs, senescence-related proteins; AMD, age-related macular degeneration; IVW, inverse-variance weighted. *P* < 0.05 was considered nominally significant and *P* < 0.00045 was considered statistically significant.

The list of 110 SRPs was in Table 1. In the discovery stage, based on MR results, 27 SRPs were suggestively associated and 7 SRPs were significantly associated with risk of AMD (Figures 4, 5; Supplementary Table 3). The 7 SRPs significantly associated with AMD risk were: C3b (odds ratio [OR] = 6.66, 95% confidence interval [CI]: 2.71–16.37, IVW-P = 3.60E-5), CD9 (OR = 2.51, 95% CI: 1.62–3.88, IVW-P = 3.88E-5), CTNNB1 (OR = 1.91, 95% CI: 1.37–2.66, IVW-P = 1.52E-4), CCL24 (OR = 4.27, 95% CI: 1.72–10.56, IVW-P = 1.69E-4), CCL1 (OR = 1.95, 95% CI: 1.37–2.76, IVW-P = 1.76E-4), CCL3L1 (OR = 1.86, 95% CI: 1.3–2.66, IVW-P = 4.13E-4), PECAM1 (OR = 2.4, 95% CI: 1.45–3.97, IVW-P = 4.21E-4). The MR-Egger intercept test and Cochran’s Q test all suggested no apparent heterogeneity or pleiotropy in these 7 results (Heterogeneity-*P* > 0.05 and Pleiotropy-*P* > 0.05).

**TABLE 1:** The 110 senescence-related proteins.

Protein	Full name	Category
CTSB	Cathepsin B	Metallo-proteases
HGF	Hepatocyte growth factor	Metallo-proteases
MMP1	Interstitial collagenase	Metallo-proteases
MMP10	Stromelysin-2	Metallo-proteases
MMP12	Macrophage metalloelastase	Metallo-proteases
MMP13	Collagenase 3	Metallo-proteases
MMP14	Matrix metalloproteinase-14	Metallo-proteases
MMP2	72 kDa type IV collagenase	Metallo-proteases
MMP3	Stromelysin-1	Metallo-proteases
MMP9	Matrix metalloproteinase-9	Metallo-proteases
PAPPA	Pappalysin-1	Metallo-proteases
PLAT	Tissue-type plasminogen activator	Metallo-proteases
PLAU	Urokinase-type plasminogen activator	Metallo-proteases
CCL1	C-C motif chemokine 1	Cytokine/Chemokine
CCL13	C-C motif chemokine 13	Cytokine/Chemokine
CCL16	C-C motif chemokine 16	Cytokine/Chemokine
CCL2	C-C motif chemokine 2	Cytokine/Chemokine
CCL20	C-C motif chemokine 20	Cytokine/Chemokine
CCL24	C-C motif chemokine 24	Cytokine/Chemokine
CCL26	C-C motif chemokine 26	Cytokine/Chemokine
CCL3	C-C motif chemokine 3	Cytokine/Chemokine
CCL3L1	C-C motif chemokine 3-like 1	Cytokine/Chemokine
CCL4L1	C-C motif chemokine 4-like	Cytokine/Chemokine
CCL5	C-C motif chemokine 5	Cytokine/Chemokine
CCL7	C-C motif chemokine 7	Cytokine/Chemokine
CCL8	C-C motif chemokine 8	Cytokine/Chemokine
CSF1	Macrophage colony-stimulating factor 1	Cytokine/Chemokine
CSF2	Granulocyte-macrophage colony-stimulating factor	Cytokine/Chemokine
CXCL1	Growth-regulated alpha protein	Cytokine/Chemokine
CXCL10	C-X-C motif chemokine 10	Cytokine/Chemokine
CXCL12	Stromal cell-derived factor 1	Cytokine/Chemokine
CXCL16	C-X-C motif chemokine 16	Cytokine/Chemokine
CXCL8	Interleukin-8	Cytokine/Chemokine
IL10	Interleukin-10	Cytokine/Chemokine
IL13	Interleukin-13	Cytokine/Chemokine
IL15	Interleukin-15	Cytokine/Chemokine
IL18	Interleukin-18	Cytokine/Chemokine
IL1A	Interleukin-1 alpha	Cytokine/Chemokine
IL1B	Interleukin-1 beta	Cytokine/Chemokine
IL2	Interleukin-2	Cytokine/Chemokine
IL32	Interleukin-32	Cytokine/Chemokine
IL6	Interleukin-6	Cytokine/Chemokine
IL7	Interleukin-7	Cytokine/Chemokine
SPP1	Osteopontin	Cytokine/Chemokine
TNF	Tumor necrosis factor	Cytokine/Chemokine
AREG	Amphiregulin	Growth factor
BMP2	Bone morphogenetic protein 2	Growth factor
BMP6	Bone morphogenetic protein 6	Growth factor
EREG	Epiregulin	Growth factor
FGF1	Fibroblast growth factor 1	Growth factor
FGF2	Fibroblast growth factor 2	Growth factor
FGF7	Fibroblast growth factor 7	Growth factor
GDF15	Growth/differentiation factor 15	Growth factor
IGF1	Insulin-like growth factor I	Growth factor
KITLG	Kit ligand	Growth factor
NRG1	Neuregulin-1	Growth factor
PGF	Placenta growth factor	Growth factor
VEGFA	Vascular endothelial growth factor A	Growth factor
VEGFC	Vascular endothelial growth factor C	Growth factor
ANGPT1	Angiopoietin-1	Intercellular signal molecule
ANGPTL4	Angiopoietin-related protein 4	Intercellular signal molecule
DKK1	Dickkopf-related protein 1	Intercellular signal molecule
EDN1	Endothelin-1	Intercellular signal molecule
ESM1	Endothelial cell-specific molecule 1	Intercellular signal molecule
GMFG	Glia maturation factor gamma	Intercellular signal molecule
ANG	Angiogenin	Miscellaneous
CD55	Complement decay-accelerating factor	Miscellaneous
GEM	GTP-binding protein GEM	Miscellaneous
ICAM1	Intercellular adhesion molecule 1	Miscellaneous
ICAM3	Intercellular adhesion molecule 3	Miscellaneous
IGFBP7	Insulin-like growth factor-binding protein 7	Miscellaneous
LCP1	Plastin-2	Miscellaneous
NAP1L4	Nucleosome assembly protein 1-like 4	Miscellaneous
PECAM1	Platelet endothelial cell adhesion molecule	Miscellaneous
C3_C3	Complement C3	Protease inhibitors
C3_C3a	Complement C3a	Protease inhibitors
C3_C3b	Complement C3b	Protease inhibitors
C3_C3d	Complement C3d	Protease inhibitors
C3_iC3b	Complement inactivated C3b	Protease inhibitors
CST4	Cystatin-S	Protease inhibitors
IGFBP1	Insulin-like growth factor-binding protein 1	Protease inhibitors
IGFBP2	Insulin-like growth factor-binding protein 2	Protease inhibitors
IGFBP3	Insulin-like growth factor-binding protein 3	Protease inhibitors
IGFBP4	Insulin-like growth factor-binding protein 4	Protease inhibitors
IGFBP5	Insulin-like growth factor-binding protein 5	Protease inhibitors
IGFBP6	Insulin-like growth factor-binding protein 6	Protease inhibitors
SERPINB4	Serpin B4	Protease inhibitors
SERPINE1	Plasminogen activator inhibitor 1	Protease inhibitors
SERPINE2	Glia-derived nexin	Protease inhibitors
TIMP2	Metalloproteinase inhibitor 2	Protease inhibitors
ITPKA	Inositol-trisphosphate 3-kinase A	Protein modifying enzymes
MIF	Macrophage migration inhibitory factor	Protein modifying enzymes
CTNNB1	Catenin beta-1	Transcription factors and regulators
ETS2	Protein C-ets-2	Transcription factors and regulators
HMGB1	High mobility group protein B1	Transcription factors and regulators
JUN	Transcription factor Jun	Transcription factors and regulators
ACVR1B	Activin receptor type-1B	Transmembrane signal receptors
AXL	Tyrosine-protein kinase receptor UFO	Transmembrane signal receptors
CD9	CD9 antigen	Transmembrane signal receptors
CSF2RB	Cytokine receptor common subunit beta	Transmembrane signal receptors
EGF	Epidermal growth factor	Transmembrane signal receptors
EGFR	Epidermal growth factor receptor	Transmembrane signal receptors
FAS	Tumor necrosis factor receptor superfamily member 6	Transmembrane signal receptors
IL6ST	Interleukin-6 receptor subunit beta	Transmembrane signal receptors
ITGA2	Integrin alpha-2	Transmembrane signal receptors
PLAUR	Urokinase plasminogen activator surface receptor	Transmembrane signal receptors
SELPLG	P-selectin glycoprotein ligand 1	Transmembrane signal receptors
TNFRSF11B	Tumor necrosis factor receptor superfamily member 11B	Transmembrane signal receptors
TNFRSF1A	Tumor necrosis factor receptor superfamily member 1A	Transmembrane signal receptors
TNFRSF1B	Tumor necrosis factor receptor superfamily member 1B	Transmembrane signal receptors

### 3.2 MR results in the validation stage

**FIGURE 6:**
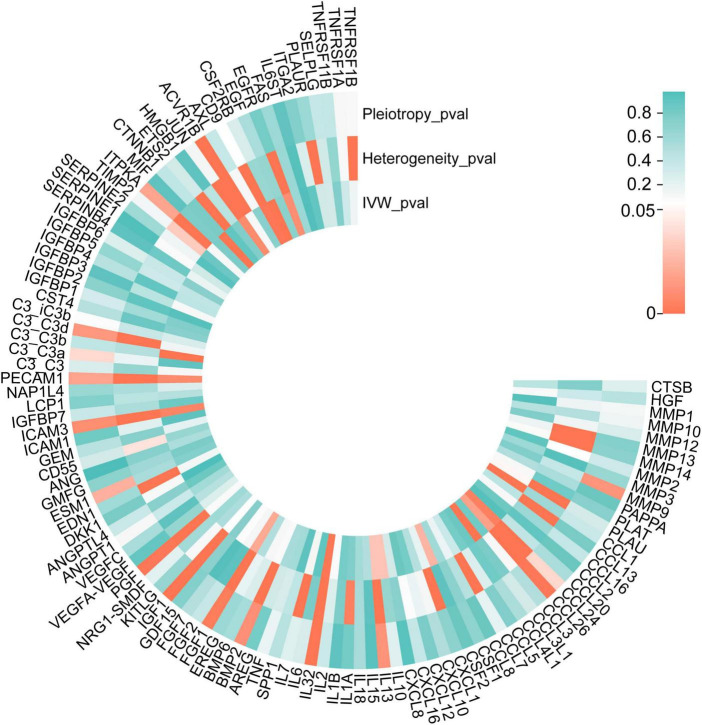
*P*-values of IVW, heterogeneity test and pleiotropy test of SRPs and AMD risk in validation stage. MR: Mendelian randomization; SRPs, senescence-related proteins; AMD, age-related macular degeneration; IVW, inverse-variance weighted.

**FIGURE 7:**
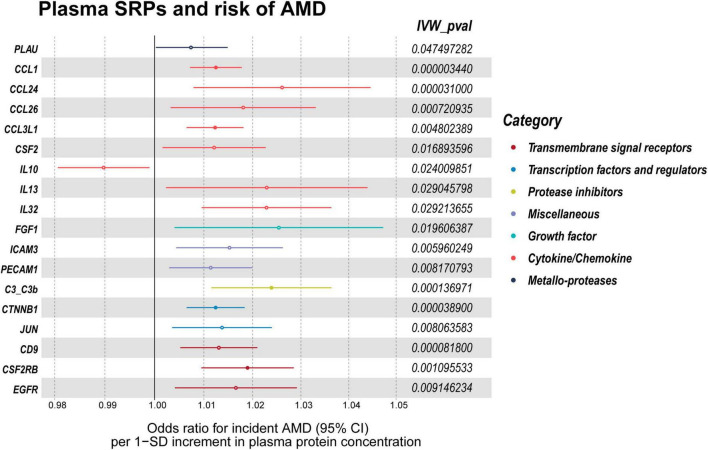
Forest plot of the MR results in validation stage. MR: Mendelian randomization; SRPs, senescence-related proteins; AMD, age-related macular degeneration; IVW, inverse-variance weighted. *P* < 0.05 was considered nominally significant and *P* < 0.00045 was considered statistically significant.

In the validation stage, based on MR results, 18 SRPs were suggestively associated and 5 SRPs were significantly associated with risk of AMD (Figures 6, 7; Supplementary Table 4). The 5 SRPs significantly associated with AMD risk were: CCL1 (OR = 1.012, 95% CI: 1.007–1.018, IVW-P = 3.44E-6), CCL3L1 (OR = 1.012, 95% CI: 1.006–1.018, IVW-P = 3.1E-5), CTNNB1 (OR = 1.012, 95% CI: 1.006–1.018, IVW-P = 3.89E-5), CSF2RB (OR = 1.019, 95% CI: 1.009–1.029, IVW-P = 8.18E-5), C3b (OR = 1.024, 95% CI: 1.012–1.036, IVW-P = 1.37E-4). The MR-Egger intercept test and Cochran’s Q test also suggested no apparent heterogeneity or pleiotropy in these 5 results (Heterogeneity-*P* > 0.05 and Pleiotropy-*P* > 0.05). Notably, the effect of C3b, CCL1, CCL3L1, and CTNNB1 on the risk of AMD was validated in this stage. The related Gene Ontology (GO) annotations and related diseases of these 4 proteins were showed in Table 2.

**TABLE 2:** The 4 SRPs significantly associated with AMD risk.

Protein	Related GO annotations	Related diseases
CCL1	GO:0090026 positive regulation of monocyte chemotaxis; GO:0048245 eosinophil chemotaxis; GO:0032740 positive regulation of interleukin-17 production.	Asthma, allergic rhinitis, rheumatoid arthritis, and multiple sclerosis
CCL3L1	GO:0048245 eosinophil chemotaxis; GO:0072677 eosinophil migration; GO:0002548 monocyte chemotaxis.	HIV infection/AIDS, rheumatoid arthritis
C3b	GO:0001970 positive regulation of activation of membrane attack complex; GO:0150064 vertebrate eye-specific patterning; GO:0001798 positive regulation of type IIa hypersensitivity.	Atypical hemolytic uremic syndrome (aHUS) and AMD
CTNNB1	GO:0007403 glial cell fate determination; GO:0044336 canonical Wnt signaling pathway involved in negative regulation of apoptotic process; GO:0061324 canonical Wnt signaling pathway involved in positive regulation of cardiac outflow tract cell proliferation.	Several types of cancer, including uveal melanoma, colorectal cancer, and ovarian cancer

SRPs, senescence-related proteins; AMD, age-related macular degeneration; GO, Gene Ontology.

## 4 Discussion

Age-related macular degeneration is a neurodegenerative disease predominantly affecting the elders, which can cause vision loss and has a significant impact on the quality of life of affected individuals (Wood et al., 2011). Identifying systemic risk factors for AMD is important for preventing development of this disease. Therefore, this study aimed to explore the relationship between plasma SRPs and AMD risk by two-sample MR analysis, which is a useful tool for assessing and determining clinically associated characters for multiple diseases (Davey Smith and Hemani, 2014). As a results, high plasma levels of 4 SRPs (C3b, CCL1, CCL3L1, and CTNNB1) were identified to be associated with increased risk of AMD.

Blood supply of the retina consists of retinal microcirculatory system and the underlying choriocapillaris (Funk, 1997). Notably, systemic inflammation has been associated with the development of AMD (Sannan, 2023). In this study, the identified 4 SRPs associated with AMD risk are tightly associated with inflammation processes, which further highlighted the important role of systemic inflammation and the high plasma levels of inflammatory mediators in the pathogenesis of AMD.

Complement C3 is a gene that plays a crucial role in the activation of the complement cascade and major effector functions of complement are mediated through C3b (Haapasalo and Meri, 2019). In AMD, the injury of blood-retinal barrier allows leakage of serum proteins, including complement components, into the retina from the underlying choriocapillaris (Katschke et al., 2018). Local complement activation leads to the recruitment of microglia into the lesion, which then produce additional complement components, prune complement-coated synapses away from neurons, depriving neurons of trophic support, and cause neuroinflammation that adds to neuronal damage and loss (Stephan et al., 2012). The elevated level of plasma C3b may promote the development of AMD by facilitating the neuroinflammation in the retina, which explained the observed association between higher plasma level of C3b and the increased risk of AMD. Notably, C3b has been associated with AMD (Helgason et al., 2013) and taken as the therapeutic target for AMD (Yang et al., 2022; Jia et al., 2023). Yang et al. (2022) reported the preclinical assessment and phase 1 clinical outcomes of a bispecific fusion protein (efdamrofusp alfa), which is capable of neutralizing both VEGF isoforms and C3b/C4b, in neovascular AMD (nAMD) treatment. However, recent studies focused on neutralizing local C3b instead of lowering circulating level of it. Considering the identified causal effect of C3b on AMD risk in this study, circulating C3b lowering strategy might be a potential way for reducing AMD risk, which calls for further studies.

The other three AMD-associated proteins identified in this study may also contribute to the development of AMD through the induction of neuroinflammation in the retina. CCL1 belongs to the C-C subfamily of chemokines, which are secreted proteins involved in immunoregulatory and inflammatory processes. It binds to the C-C motif receptor 8 (CCR8). In addition, it has been implicated in various inflammation-associated diseases, including asthma (Hurme et al., 2022), rheumatoid arthritis (White et al., 2013), and multiple sclerosis (Schropp et al., 2023). In asthma, CCL1 is thought to play a role in recruiting inflammatory cells to the airways, leading to airway inflammation and hyperresponsiveness. Targeted drug discovery efforts have focused on developing drugs that can block the interaction between CCL1 and its receptor, with the aim of reducing inflammation and disease progression (Connolly et al., 2012). CCL3L1 encodes a protein that binds to several chemokine receptors, including CCR5 (Urban et al., 2009). CCL3L1 has also been implicated in inflammatory diseases such as rheumatoid arthritis (Nordang et al., 2012). CTNNB1 encodes a protein that is part of a complex of proteins that make up adherens junctions (AJs) (van der Wal and van Amerongen, 2020), which are essential for creating and maintaining epithelial cell layers by regulating cell growth and adhesion between cells. In addition, CTNNB1 is related to cholesterol homeostasis (Chen et al., 2023), which might also be the mechanical basis of the association between plasma CTNNB1 level and AMD risk.

There were some limitations in this MR-designed investigation, despite its many advantages over conventional epidemiological studies. First, this study only included European-ancestry individuals, which suggests that our findings cannot be directly applied to other populations. Second, our findings only revealed the causality between plasma levels of several SRPs and AMD, not the underlying mechanisms, which call for further researches.

We obtained druggable genes from the DGIdb database (Freshour et al., 2021). Importantly, most of the SRGs are druggable genes, including the four genes identified as risk factors for AMD, i.e., C3b, CCL1, CCL3L1, and CTNNB1. Therefore, our study may provide some novel potential drug targets for AMD or the rationale of existing drugs. Still, the results need to be confirmed by further studies and it is hoped that these 4 SRPs will serve as new drug targets for preventing the incidence of AMD.

In conclusion, we provided the genetic evidence that plasma levels of C3b, CTNNB1, CCL1, and CCL3L1 are causally associated with risk of AMD, which highlights the role of systemic inflammation in the pathophysiology of AMD. Given that the genes encoding these four proteins are all druggable targets, the findings may contribute to understanding the pathogenesis of AMD and the development of new therapeutic or preventive strategy for AMD.

## Data availability statement

The original contributions presented in this study are included in this article/Supplementary material, further inquiries can be directed to the corresponding author.

## Ethics statement

Human subjects or animal subjects were not included in this study. This study used only publicly available, deidentified data from previously published works, making it exempt according to the Wuhan Fourth Hospital Institutional Review Board. Our research adhered to the tenets of the Declaration of Helsinki.

## Author contributions

SL: Conceptualization, Data curation, Formal analysis, Investigation, Methodology, Visualization, Writing—original draft. MH: Conceptualization, Data curation, Formal analysis, Methodology, Writing—original draft. ZW: Conceptualization, Methodology, Supervision, Writing—review and editing.

## Footnotes

1
https://www.dgidb.org/
